# Household food insecurity and physically demanding work during pregnancy are risk factors for low birth weight in north Shewa zone public hospitals, Central Ethiopia, 2021: a multicenter cross-sectional study

**DOI:** 10.1186/s12887-022-03480-2

**Published:** 2022-07-14

**Authors:** Elias Yadeta Debele, Merga Dheresa, Dawit Tamiru, Tegenu Balcha Wadajo, Kasiye Shiferaw, Lemesa Abdisa Sori, Nega Assefa

**Affiliations:** School of Nursing and Midwifery, College of Health and Medical Sciences, Haramaya University, P.O. BOX 138, Harar, Ethiopia

**Keywords:** Central Ethiopia, Food insecurity, Low birth weight, Physically demanding work during pregnancy

## Abstract

**Background:**

Despite numerous efforts to improve the quality of maternal and child health medical services, over 20 million babies are born with low birth weights each year globally. However, factors related to low birth weight like physically demanding work during pregnancy, intimate partner violence, and food insecurity have not been explored well in Ethiopia. Thus, this study aimed to assess the prevalence of low birth weight and associated factors among neonates born in public Hospitals in North Shewa Zone, Central Ethiopia.

**Methods:**

A hospital-based cross-sectional study design was conducted from June 15 –to July 15, 2021, in North Shewa public hospitals. A total of 441 mothers and newborn pairs were selected by systematic random sampling. Data were collected using a pretested and structured interviewer-administered questionnaire with chart reviewing. Data entry and analysis were done using Epi Data version 3.1 and Statistical Package for the Social Sciences version 26 respectively. Binary logistic regression was done to identify factors associated with low birth weight. Adjusted odds ratio with its 95% confidence interval and a *p*-value less than 0.05 was considered to declare the statistically significant association.

**Results:**

The prevalence of low-birth-weight was 17.7% (95% CI: 14.3, 21.5). Pregnancy-related complication [AOR = 2.16; 95% CI:(1.12,4.18)], grand-multiparty [AOR = 2.57; 95% CI:(1.12,5.88)], physically demanding work during pregnancy [AOR = 2.19; 95% CI:(1.11,4.33)], midd-upper arm circumference less than 23 cm [AOR = 2.54; 95% CI:(1.26,5.10)], partner violence during pregnancy [AOR = 3.77; 95% CI:(1.81,7.88)], and being member of household with food insecure [AOR = 2.31; 95% CI:(1.12,4.75)] were factors significantly associated with low birth weight.

**Conclusions:**

This study showed that the magnitude of low birth weight was relatively high. Women with pregnancy-related complications, grand multiparty, physically demanding work during pregnancy, intimate partner violence, mid-upper arm circumference less than 23 cm, and food insecurity should be prioritized for mitigating LBW. Health care professionals should focus on Screening pregnant women for intimate partner violence, physically demanding activities, undernutrition and providing appropriate treatment during all maternal continuum of care might be helpful.

**Supplementary Information:**

The online version contains supplementary material available at 10.1186/s12887-022-03480-2.

## Introduction

Low birth weight (LBW) is defined as “the weight at birth less than 2500 g (5.5lb) regardless of gestational age” According to World Health Organization [1]. It is a key predictor of the past and current public health status of the mothers like long-term maternal malnutrition, chronic maternal illness, and poor antenatal care [2]. Globally, the prevalence of LBW was estimated to be between 15.5 and 20% [3]. According to the 2019 UNICEF report, low and middle-income countries had the largest burden of LBW (97%). For example, South Asia had 28%, Sub-Saharan Africa had 13%, and Latin America and the Caribbean had 13% respectively [3].

Despite improvement in maternal and child health care, more than 4 million babies’ death occurs globally during their first 4 weeks of life with LBW being a major indirect cause of neonatal mortality. LBW newborns account for more than 80% of all neonatal deaths worldwide [4, 5]. In addition to mortality and morbidity, caring for LBW newborns imposes a considerable burden or expenses to the family, hospital, government and society as a well [6]. Furthermore, LBW increases the risk of many labor complications such as irregular heart rate patterns during labor, low Apgar scores < 7 at both first and fifth minutes, risk of sudden cardiac death in the young people and risk of elective cesarean delivery or labour induction [7, 8].

During the 65th World Health Assembly, member states of the United Nations set a national objective to reduce LBW by 30% in 2025 [3]. According to a recent report released by the World Health Organization (WHO), slow progress in the reduction of LBW has impeded the global efforts to prevent unnecessary newborn death and reduce the number of children suffering from wasting and stunting [3, 9]. Between 2000 and 2015, no region reported a significant decline in LBW prevalence; rather, both developed and developing countries recorded an increment in its magnitude. For instance, the number of LBW among live births in Sub-Saharan Africa increased from 4.4 million to 5 million between 2000 and 2015 [4]. This figure indicates that the LBW target of 2025 will unlikely to be achieved if the current trend is allowed to continue [3].

Even though the Ethiopian government took significant steps to improve maternal and child health during the Millennium Development Goals (MDG) [10] through developing and implementing national and international child health intervention strategies, for instance, the newborn and child survival strategy and the Ethiopian Pediatric Society in conjunction with UNICEF have been working in improving child health [11]. However, LBW is continuing to be among the top causes of neonatal mortality in Ethiopia [12]. EDHS report also showed neonatal mortality has increased slightly from 29 in 2016 to 33 deaths per 1000 live births in 2019. This report indicates that reaching the 2030 Sustainable Development Goal will be challenging if the current trends are kept to be continued [13, 14].

Various studies revealed that maternal nutrition, obstetric factors, chronic medical illnesses such as cardiovascular disease and HIV/AIDS, intimate partner violence, physically demanding work during pregnancy, maternal behavior and lifestyle factors, socioeconomic factors, and pregnancy-related complications are risk factors for LBW [15–18]. Women in developing countries or poor communities are at higher risk to have LBW babies. For instance, they are more likely to participate in risk factors such as physically demanding work at home and on farms, which raises the risk of having unfavorable birth outcomes like LBW [19].

Although many studies have been conducted in developed and developing countries regarding the relationship between physically demanding work during pregnancy and LBW, there has been no study in Ethiopia linking physically demanding work during pregnancy with LBW. In addition, the existing LBW research failed to consider contributing variables like intimate partner violence and household food insecurity [20–23]. Therefore, this study aimed to assess the magnitude and factors associated with LBW among neonates born in public Hospitals in North Shewa Zone, Central Ethiopia.

## Methods and materials

### Study setting and period

This study was conducted at public hospitals in North Shewa Zone. North Shewa zone is one of the 20 zones in Oromia Regional State and its administrative town is Fiche town, which is located 112 km away from Addis Ababa, the capital city of Ethiopia. The zone is administratively divided into 13 districts and two town administrations. As per the 2021 census, the zone has a total population of 1,786,067, of whom 876,252 were men and 909,815 were women [24]. In terms of health facilities, the North Shewa zone has five public hospitals, 63 health centres, and 267 health posts [25]. These Health facilities provide multidimensional health care services for the catchment’s area population. The study was conducted from June 15 –to July 30/ 2021.

### Study design and population

A public hospitals-based cross-sectional study was done among 448 mothers with their newborns who were selected by systematic random sampling technique. All alive newborns with their mothers who gave birth in selected Public Hospitals in North Shewa Zone from June 15, 20,201, to July 30, 2021, were the study population. Newborns of mothers in critical medical conditions and newborns with visible congenital anomalies were excluded from the study.

### Sample size and sampling technique

The sample size was determined for both objectives separately and a 10% non-response rate was added. The adequate sample size was obtained using a single population proportion formula with the assumptions of, a 21.6% prevalence of LBW [26], 95% confidence interval (CI), 4% margin of error, and adding a 10% non-response rate, the final sample size became 448. The average total births during the study period were approximately 1080 as estimated from the preceding months’ delivery flow of each hospital. The sampling interval was estimated by dividing the total study population of 1080 by the sample size (*n* = 448). The sampling fraction or K-value was 2. The first study participant was selected by lottery method for each hospital independently and the next participants were selected every other.

### Sampling procedures

**Fig. 1:**
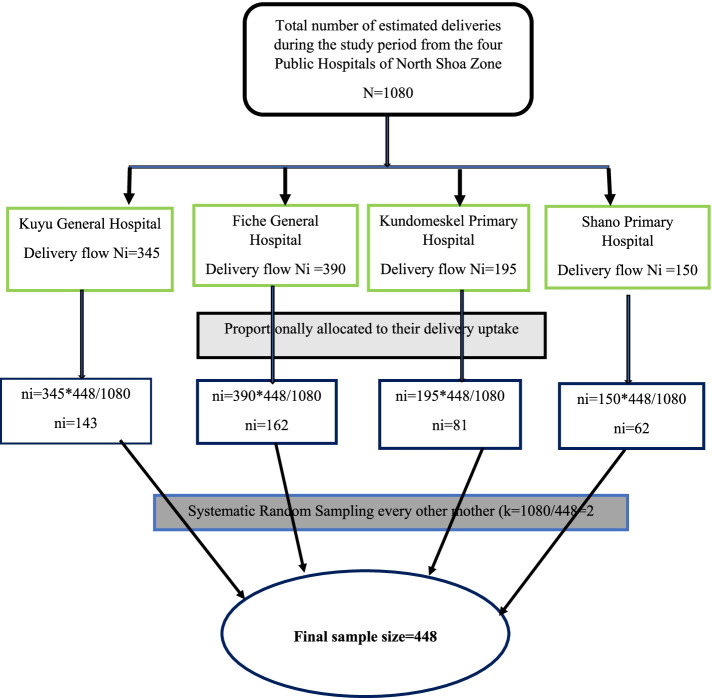
Schematic representation of the sampling procedure among newborns delivered in North Shewa zone public hospitals, Central Ethiopia, 2021

Four public hospitals from the North Shewa zone were included in the study. The number of study participants was proportionally allocated to each respective hospital based on estimations obtained from the previous delivery report as indicated in the figure below (Fig. 1).

### Data collection tools and procedures

A pretested and structured interviewer-administered questionnaire and medical record extraction were used. The questionnaire was adapted and modified from other peer-reviewed articles [6, 21, 27–31]. The questionnaire comprises different sections like women’s socio-demographic characteristics, obstetric-related factors, nutritional-related factors, intimate partner violence, household food insecurity, neonatal, and maternal behavior-related factors. The data were collected by four BSc midwives and two BSc nurses through face to face interviews and variables like neonatal birth weight, pregnancy complication, maternal hemoglobin, and gestational age were extracted from the delivery registration book and mother’s card. The Maternal-Upper Arm Circumference (MUAC) was measured on the left arm using a non-stretchable standard tape.

### Variables and measurements

**Intimate Partner Violence during Pregnancy (IPVP**)**:** was measured using a standardized tool developed by WHO [32]. women who replied “yes” to at least one of the 13 questions related to sexual, psychological, and physical violence were coded as “having experienced IPVP, whereas women who answered “no” to all of the questions were coded as not exposed to IPVP [32, 33].

**Household food insecurity:** was assessed using a Household Food Insecurity Access Scale (HFIAS) developed by Food and Nutritional Technical Assistance (FANTA) [31]. The tools were tested and validated in Ethiopia with Cronbach’s alpha value of 0.85 for both rural and urban samples [34]. The tools consist of nine items with a (Yes or No) response. All “Yes” replies were given a score of one, while “No” responses were given a score of zero. Finally, all responses were added together and participants who scored > 2 affirmative responses were considered as from a household’s food insecure whereas those who replied ≤2 affirmative responses were from a household’s food secure [31].

**Physically demanding work during pregnancy:** was measured using the following eleven items related to domestic and other activities performed during the pregnancy period with Yes or No responses [15, 30, 35]. Daily household chores, fetching water with large buckets, lifting heavy loads (> 20 kg), chopping woods, cleaning the land, planting seeds, cutting grass for cattle feeding, washing clothes/utensils for long periods, standing for longer hours (> 3 hr), squatting during routine daily activity, and bathing and milking cattle. The sum of scores ranging from 0 to 11, which further classified into two categories; participants who replied ≤3 affirmative responses were coded as not engaged in physically demanding work whereas those who replied ≥4 positive responses were coded as engaged in physically demanding work 4–11 [15, 30]. In this study, the internal consistency of physically demanding work items was (Cronbach’s alpha = 0.82).

**Daily household chores:** Whether the mother did her housework alone or with the assistance of a relative person throughout her pregnancy period.

**Undernutrition:** Mothers with mid-upper arm circumference (MUAC) < 23 cm [36].

**Low birth weight**: Newborns who weighed less than 2500 g [1]. It coded as “1” for LBW whereas “0” for others since it was an outcome variable for this study.

**Birth-to-pregnancy interval** is the period between the start of the index pregnancy and the preceding live birth. It has three categories, these are: <24monthes, between 24-47monthes, and ≥ 48 months are the three categories [37]. It was estimated by subtracting the duration of the current pregnancy from the period between the preceding childbirth and the current birth.

**Alcohol use:** use of any amount unit of alcohol whether it is locally manufactured drinks (Tela, Teje, Areka), or beer, wine, and any alcoholic-liquors beverage**s** [18].

### Data processing and analysis

The data were cleaned, checked, coded, and entered into Epi data statistical software version 3.1 and then exported to SPSS version 26 for analysis. Simple frequency, and summary statistics such as median, and interquartile range were generated as descriptive statistical analysis. The results were presented using frequencies, tables, and figures.

Bivariable and multivariable binary logistic regression analyses were performed to see the association between independent variables and the outcome. To control all possible confounders, variables which have a *P*-value < 0.25 in the bivariable analysis were retained for the final model multivariable analysis. The multi co-linearity test was also done using VIF and tolerance tests to see the presence of correlation between independent variables. However, no variables with VIF > 10 and tolerance test < 0.1 were found. The Hosmer-Lemeshow and the Omnibus test were used to test the model’s goodness of fit. The model was deemed to be a good fit since the result was found to be insignificant for the Hosmer-Lemeshow test (*p* = 0.616), but significant for the Omnibus test (*p* = 0.000).

In multivariable analysis, a *P*-value less than 0.05 was considered to declare a statistically significant association. The strength and direction of statistical association were reported using an adjusted odds ratio with it is 95% CI.

### Data quality control

The questionnaire was evaluated by experts in the related field. It was first developed in English language, then translated into Afaan Oromo and Amharic, with re-translation into English to ensure it is consistency. A pre-test was conducted in Chancho primary Hospital, on 5% of the total sample size to check for language clarity, estimate the time required for the interview, and necessary amendments were done accordingly. The training was given to data collectors and supervisors on the study’s objective, ethical principles, sample procedure, questionnaire content, confidentiality, and respondent rights. The principal investigator together with the supervisor checked the data for completeness on daily basis.

## Results

### Socio-demographic characteristics

In this study, of 448 participants, 441 delivered mothers-newborn pairs participated in the study with a 98.4% response rate. Seven respondents declined to take part in the study. The median and interquartile range (±IQR) of mothers’ age was 25 (±7) ranging from 18 to 41. Slightly more than two-thirds, 305 (69%) of the mothers were in the age group of 21–34 years. The majority of the participants (93.2%) were married and more than three-quarters, 370 (83.9%) were Orthodox Christians. Regarding maternal occupational status, more than half (59.9%) of the neonate’s mothers were housewives. The median and interquartile range of household incomes of the respondents were 3000 ETB ± 1500 per month (Table 1).

**Table 1:** Sociodemographic characteristics of study participants at public hospitals in North Shewa Zone, Oromia Region, central Ethiopia, June 15 to July 30, 2021(*n* = 441)

Variable	Frequency	Percentage
Age		
≤ 20 years	87	19.7
21–34 years	305	69.2
> 34 years	49	11.1
Religion		
Orthodox	370	83.9
Protestant	57	12.9
Others^a^	14	3.1
Marital status		
Married	411	93.2
Divorced	21	4.8
Others^b^	9	2.0
Residence		
Urban	168	38.1
Rural	273	61.9
Level of mother’s education		
No education	165	37.4
Primary education	143	32.4
Secondary education	98	22.2
Higher education	35	7.9
Occupational status of the mothers		
Student	41	9.3
Housewife	264	59.9
Private employee	75	17
Government employee	29	6.6
Merchant	32	7.3
Level of husband’s education		
No education	79	17.9
Primary education	123	27.9
Secondary education	143	32.4
Tertiary education	96	21.8
Occupational status of husbands		
Private employee	107	24.3
Government employee	99	22.4
Farmer	185	42.0
Others ^c^	49	11.1
Monthly income in ETB		
< 1000	25	5.7
1000–2000	58	13.2
2001–4000	255	57.8
> 4000ETB	103	23.4
Sex of newborns		
Male	232	52.6
Female	209	47.4
Distance to the health facility		
< 1 hr	206	46.7
≥ 1 hr	235	53.3
Family size		
≤ 5	312	70.7
> 5	129	29.3

*ETB* Ethiopian Birr; *hr* hour; ^a^, Muslim, Wakefata; ^b^, Single, Widowed; ^c^student, Merchant

### Maternal and child health, and obstetric characteristics

Of the total study participants, 365 (82.2%) had attended ANC follow-up for their last pregnancies. In terms of pregnancy intentions, slightly more than three fourth (77.8%) of the neonate’s mothers desired to have the current pregnancy. Nearly a quarter of the mothers, 68 (22.8%), gave birth to the present newborns within less than 24 months after the previous childbirth. Concerning the mode of delivery, 338 (76.6%) of mothers had their babies via spontaneous vaginal delivery. In terms of gestational age at birth, 391 (88.7%) of mothers delivered their babies at full term (Table 2).

**Table 2:** Obstetric related characteristics of mothers who gave birth at Public Hospitals in North Shewa Zone, Oromia Region, central Ethiopia, June 15 to July 30, 2021(*n* = 441)

Variables	Frequency	Percentage
ANC follow up		
Yes	365	82.8
No	76	17.2
Number of ANC visits (*n* = 365)		
≤ three	250	68.6
Four and above	115	31.4
Time of starting ANC visit (*n* = 365)		
≤ 16 weeks	240	65.8
> 16 weeks	125	34.2
Parity		
Prim parous	129	29.3
Multiparous	242	54.9
Grand multiparous	70	15.9
Type of pregnancy		
Singleton	429	97.3
Multiple	12	2.7
Birth interval (*n* = 312)		
< 24 months	68	21.8
24–48 months	159	50.0
> 48 months	85	27.2
Pregnancy complications		
Yes	107	24.3
No	334	75.7
Mode of deliveries		
SVD	338	76.6
C/S	74	14.0
Instrumental delivery	29	6.6
Gestational age at delivery		
Term	391	88.7
Preterm	50	11.3
Type of Pregnancy		
Planned and wanted	343	77.8
Unplanned but wanted	85	19.3
Unplanned and unwanted	13	2.9

*ANC* Antenatal Care; *C/S* Cesarean Section; *SVD* Spontaneous Vaginal Delivery

### Pregnancy-related complications

**Fig. 2:**
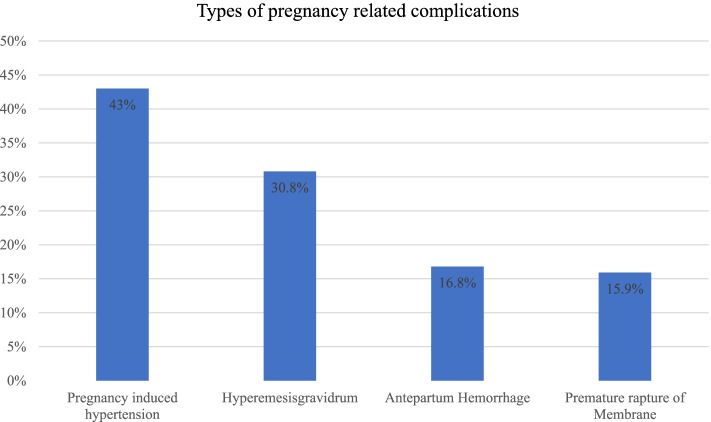
Pregnancy-related complications among mothers who gave birth in North Shewa zone public hospitals, central Ethiopia, June 15 to July 30, 2021 (*n* = 107)

Regarding pregnancy-related complications during pregnancy, among 441 mothers, 107 (24.3%) encountered pregnancy complications. The commonest complications were pregnancy-induced hypertension, severe nausea and vomiting, APH, and PROM as indicated below (Fig. 2).

### Maternal nutritional and lifestyle-related characteristics

Of the total study participants, 363 (82.3%) of them were from household food secure and 79 (17.9%) had MUAC less than 23 cm (undernourished). The level of hemoglobin among neonates’ mothers ranged from 9.0 g/dl to 16.2 g/dl with a median and interquartile range (±IQR) of 13.2 g/dl (±2 g/dl). The proportion of mothers who had engaged in physically demanding work during the current pregnancy was 116 (26.3%). About three fourth, 332 (75.3%) of respondents took iron and folic acid supplements at least once and 114 (25. 9%) of them took less than 60 tablets during their recent pregnancies. 198 (44.9%) of mothers drank alcohol during their current pregnancy period (Table 3).

**Table 3:** Nutritional, lifestyle, and behavioral characteristics of mothers who gave birth at Public Hospitals in North Shewa Zone, Oromia Region, central Ethiopia, June 15 to July 30, 2021(*n* = 441)

Variables	Frequency	Percentage
Food security status		
Food secured	363	82.3
Food insecured	78	17.7
Engaged in physically demanding work		
Yes	116	26.3
No	325	73.7
Exposed to intimate partner violence		
Yes	100	22.7
No	341	77.3
MUAC		
< 23 cm	79	17.9
≥ 23 cm	362	82.1
Ever drunk alcohol during the current pregnancy		
Yes	198	44.9
No	243	55.1
Took iron tablets during the current pregnancy		
No	109	24.7
< 60	114	25.9
60–90	218	49.4
Have you been exposed to people smoking in the surroundings		
Yes	62	14.1
No	379	85.9
Did you take an additional diet during the current pregnancy		
Yes	214	48.5
No	227	51.5
Have you counselled on nutrition during the current pregnancy		
Yes	269	61.0
No	172	39.0
Did you fast during the current pregnancy		
Yes	206	46.7
No	235	53.3
Have you taken herbal medicine during the current pregnancy		
Yes	49	11.1
No	392	88.9
Haemoglobin level		
< 11 g/dl	45	89.8
≥ 11 g/dl	396	10.2

*MUAC* Mid-Upper Arm Circumference; *ANC* Antenatal care

### Maternal medical history related characteristics

Of the total participants, almost 11 (2.5%) had a pre-existing medical disease. Of this four (36.3%) had chronic hypertension, four (36.3%) were reactive to HIV and two (18.1%) had DM respectively (Table 4).

**Table 4:** Maternal medical history related characteristics among mothers who gave birth at Public Hospitals in North Shewa Zone, Oromia Region, central Ethiopia, June 15 to July 30, 2021(*n* = 441)

Variables	Frequency	Percentage
Pre-existing medical condition		
Yes	11	2.5
No	430	97.5
Types of medical conditions (*n* = 11)		
Chronic hypertension	4	36.3
Diabetes Mellitus	2	18.1
Reactive to HIV	4	36.3
Others^a^	3	27.2

^a^Urinary tract infection

### The magnitude of low birth weight

Of 441 study participants, 78 had low birth weight newborns. Therefore, the magnitude of low birth in the public hospital in the North Shewa zone was 17.7% (95% CI: 14.3, 21.5). The median and interquartile range (±IQR) of the birth weight of the newborns was 3000 g (±800 g) ranging from1000 grams to 4500 g.

### Factors associated with low birth weight

Among all variables, age of the mothers, parity, gestational age at birth, pregnancy-related complication, additional meal intake, alcohol intake, ANC follow-up, hemoglobin level < 11, physically demanding work during the current pregnancy, food insecurity, MUAC less than 23 cm, and intimate partner violence were associated with LBW at *p*-value less than 0.25 in bivariable analysis. All variables with a p-value < 0.25 were retained for multivariable analysis. Accordingly, parity, pregnancy-related complications, MUAC, food insecurity, hard physical work during pregnancy, and intimate partner violence were statistically associated with LBW.

In the present study, the odds of delivering LBW among mothers from households food-insecure were 2.3 times (AOR = 2.31; 95% CI: 1.12, 4.75) higher as compared to mothers from households food-secure. Neonates who were born to grand multiparous mothers had 2.6 times (AOR = 2.57; 95% CI: 1.12, 5.88) higher odds of being LBW compared to neonates delivered from multiparous. Neonates born to mothers who were engaged in physically demanding work during the current pregnancy period had a 2.2 times higher probability of being LBW than neonates born from mothers who didn’t engage in physically demanding work (AOR = 2.19; 95% CI: 1.11, 4.33). Mothers who had MUAC less than 23 cm were 2.7 times more likely to have LBW as compared to mothers with MUAC greater than 22 cm (AOR = 2.54; 95% CI: 1.26,5.10). The likelihood of having LBW among mothers who had encountered pregnancy-related complications during index pregnancy was 2 times higher (AOR = 2.16; 95% CI: 1.12, 4.18) as compared to mothers without pregnancy-related complications. Furthermore, Neonates born from mothers who were exposed to IPVP were 3.8 times (AOR = 3.77; 95% CI: 1.81, 7.88) more likely to be LBW as compared to neonates born from mothers who did not expose to IPVP (Table 5).

**Table 5:** Bivariable and multivariable analyses to assess factors associated with LBW at public hospitals in North Shewa Zone, central Ethiopia, 2021(*N* = 441)

Variables (*n* = 441)	LBW		COR (95% CI)	AOR (95% CI)
	Yes (%)	No (%)		
Age				
≤ 20	24 (27.6)	63 (72.4)	2.68 (1.45, 4.78) *	2.39 (0.91, 6.30)
21–34	38 (12.5)	267 (87.5)	1	1
> 34	16 (32.7)	33 (67.3)	3.41(1.71, 6.77) **	1.24 (0.49, 3.15)
Parity				
Prim parous	22 (17.1)	107 (82.9)	1.64 (0.89, 3.01)	0.79 (0.31, 2.00)
Multiparous	27(11.20	215 (88.8)	1	1
Grand multiparous	29 (41.4)	41 (58.6)	5.63 (3.03, 10.49) **	**2.57 (1.12, 5.88)** *
Gestational age				
Preterm	18 (36.0)	32 (64.0)	3.10 (1.64, 5.88) *	1.41 (0.62, 3.21)
Term	60 (15.3)	331(84.7)	1	1
Pregnancy complication				
Yes	41(38.3)	66 (61.70	4.99(2.97, 8.37) **	**2.16 (1.12, 4.18)** *
No	37 (11.1)	297 (88.9)	1	1
Have you taken additional food during the current pregnancy				
Yes	29 (13.6)	185 (86.4)	1	1
No	49 (21.6)	178 (78.4)	1.76 (1.06,2.91) *	0.59 (0.30, 1.18)
Ever drunk alcohol during the current pregnancy				
Yes	59 (29.8)	139 (70.2)	5.00 (2.86, 8.75) **	1.12 (0.53, 2.38)
No	19 (7.8)	224 (92.2)	1	1
ANC follow up				
Yes	55 (15.1)	310 (84.9)	1	1
No	23 (30.3)	53 (69.7)	2.45(1.39, 4.31) *	1.67 (0.81, 3.47)
Hemoglobin level				
< 11 g/dl	21 (46.7)	24 (53.3)	5.20 (2.72, 9.96) **	1.19 (0.52, 2.74)
≥ 11 g/dl	57 (14.4)	339 (85.60	1	1
Engaged in physically demanding work during the current pregnancy				
Yes	43 (37.1)	73 (62.9)	4.88 (2.92, 8.17) **	2.19 (1.11, 4.33) *
No	35 (10.8)	290 (89.2)	1	1
Exposed to intimate partner violence				
Yes	64 (34.2)	123 (65.8)	8.92 (4.81, 16.54) **	**3.77 (1.81, 7.88)** *
No	14 (5.5)	240 (94.5)	1	1
Food security status				
Secured	39 (10.7)	324 (89.3)	1	1
In secured	39 (50)	39 (50)	8.30 (4.77, 14.46) **	**2.31 (1.12, 4.75)** *
MUAC measurement				
< 23 cm	38 (48.1)	41 (51.9)	7.46 (4.31, 12.94) **	**2.54 (1.26, 5.10)** *
≥ 23 cm	40 (11.0)	322 (89.0)	1	1

*Significant with *p*-value < 0.05 and **Significant with *p*-value < 0.001

*CI* Confidence Interval; *COR* Crude Odd Ratio; *AOR* Adjusted Odd Ratio; *MUAC* mid-upper circumference; *ANC* Antenatal Care

## Discussion

The present study revealed that the magnitude of LBW among newborns delivered at public hospitals in North Shewa was 17.7%. This finding was consistent with studies done in southern Ethiopia 18% [16], eastern Ethiopia 21.6% [38], Dessie town 15.6% [23], and Nepal 21.56% [39].

However, higher than studies conducted in southern Ethiopia 8.1% [40], northern Ethiopia 12.0% [41], Kenya 12.3% [42], Ghana 10% [43], Nepal 9.4% [44], India 13.8% [45]. This discrepancy may be due to differences in the study setting, study period, seasonal variation, and inclusion of private Hospitals in prior studies [46]. Another possible explanation could be the difference in the proportion of mothers’ neonates who received ANC in northern and southern Ethiopia (91.5 and 91.7%) respectively, compared to the current study, which found that 82.0% of mothers received at least one ANC contact. Thus, pregnant women who had ANC follow-up may receive improved disease screening and prevention, as well as better nutritional advice before giving birth.

The present proportion is, however lower than studies conducted in southern Ethiopia 34.1% [20], Uganda 25.5% [47], Ghana 29.6% [28], India 31% [48], and Nepal 23.6% [49]. This disparity might be due to differences in study time, geographical variation and lower sample size compared to the current study [47–49]. Another possible reason is that most of the previous studies were carried out in referral hospitals; where many pregnant women were referred from outlying health facilities due to various difficulties.

In this study, mothers who were living in food-insecure households were more likely to have LBW babies than mothers living in food-secure households. This result is in agreement with the studies conducted in Ethiopia, Bangladesh, and New York City [50–53]. This may be because mothers who were live in food-insecure households may lack the resources or ability to produce enough food/or generate sufficient income on a long-term basis, resulting in the mother receiving insufficient nutrients during her pregnancy, which are critical for the fetus’ growth and development, especially in the second and third trimesters. Another reason is that inadequate nutritional intake during pregnancy as a result of food scarcity combined with poor maternal health (depressive symptoms) leads to impaired placental growth, which reduces nutrient transfer from mother to the fetus [52, 53]. Therefore, intervention should be focused on mothers who live in food-insecure households.

In the present study, neonates born to grand multiparous mothers had a higher probability of being LBW. This finding was supported by studies conducted in Ethiopia and California [16, 54]. This could be explained by the fact that women with higher parities are more likely to give birth to LBW than women with lower parities because of shorter birth intervals, which impose excessive energy demands on the mother with no time for postpartum recovery [55]. Moreover, as the number of births increases, there is a higher probability of having a large family, which could have an impact on the family’s socioeconomic status [16]. These findings may encourage the use of proper family planning services to achieve adequate birth intervals and desired family size.

Mothers who had a pregnancy-related complication during their current pregnancy had a higher probability of having LBW than mothers who did not have a complication. This finding is in agreement with previous studies done in Northern Ethiopia and Kenya [42, 56, 57]. This could be explained by the fact that mothers with pregnancy-related complications like hypertension and APH were more likely to deliver LBW than mothers who did not encounter complications. This is because pregnancy-related complication results in inadequate placental perfusion, as a result, the fetus receives less nutrition and oxygen which leads to LBW or fetal death [58, 59]. Therefore, it is suggested that pregnant women should be informed of the risk indicators of pregnancy and the various causes of such issues; so that they may be diagnosed and treated as soon as possible.

In this study, the odds of being LBW was significantly increased among neonates born to mothers who were engaged in heavy physical work during their current pregnancy than neonates born to mothers who did not engage in heavy physical work. This finding is supported by finding from the studies done in Cairo, Nepal, and India [15, 30, 35]. This could be explained by the fact that an increase in the activity of the sympathetic nervous system within active muscles following strenuous work causes blood to shift from visceral arteries to active muscles and reduces perfusion of the uterus and placental arteries [60]. So, health care providers should play an important role regarding decisions on work activity restrictions and adequate rest during pregnancy.

This study also revealed that mothers with MUAC less than 23 cm were more likely to have LBW babies. This finding was comparable with the finding of studies conducted in Ethiopia and Kenya [56, 61, 62], where MUAC < 23 cm was a significant predictor of LBW. This could be because poor maternal nutrition compromises the supply of nutrients to the developing fetus as newborns solely depend on mothers’ nutritional status and placental feeding during pregnancy [63]. Therefore, it is important to ensure women should have healthy diets, adequate services, rest, micronutrient supplementation and care that is fundamental for the survival and well-being of mothers and their children.

In the present study, the odds of having LBW was higher among mothers exposed to IPVP than mothers who had not been exposed to IPVP. This result was in agreement with the result of the studies done in Ethiopia, Bangladesh, and Tanzania [16, 64, 65]. The possible justification is that prenatal exposure to IPVP may impede mothers’ healthcare-seeking behaviors, healthcare utilization, and decision-making in any aspect of their life. Furthermore, IPVP leads to persistent psychosocial stress in women, which increases the risk of LBW [66]. These findings may support to launch the WHO recommendations of healthcare providers should assess exposure to intimate partner violence when assessing other conditions that may be caused or complicated by IPVP to improve the subsequent care.

The result of the present study revealed that preterm birth was not significantly associated with low birth weight. But this finding is in contrast to the studies conducted in Tigray, Amhara, south-eastern Ethiopia, where the risk of being low birth weight babies was found to be significantly higher among newborns delivered at a gestational age of less than 37 completed weeks [21, 38, 67]. This discrepancy could be explained by the difference in sample size, study setting and study design. For instance, a study conducted in Amhara and south-eastern Ethiopia utilized a case-control study design, and a study in Tigray included 1152 babies, which was very high compared to the present studies.

### Strength and limitations of the study

This multi-centre study used primary data that was supplemented with medical record extraction, which will reduce the number of missing important factors. The study has also some limitations that should be kept in mind when interpreting the results. Because of the study design, it may not establish the cause and effect relationship. Secondly, we used self-reporting (interview response) to assess some variables like intimate partner violence and food insecurity, which may have a social desirability bias.

## Conclusion

This study revealed that newborns delivered to almost two out of every ten mothers were found to be low birth weight. Physically demanding work during pregnancy, food insecurity, intimate partner violence, grand multiparty, MUAC less than 23 cm, and pregnancy-related complications were significantly associated with LBW. Therefore, Healthcare professionals should focus on screening pregnant women for undernutrition, and exposure to partner violence and ensure that women have access to important health information about the causes of low birth weight. Public health education and awareness on the importance of limiting physically demanding activities during the pregnancy period might reduce the low birth weight.

## Supplementary Information

**Additional file 1.**

## Data Availability

The datasets used and/or analyzed during the current study are not publicly available due to the privacy of the participants and institutional restrictions but are available from the corresponding author upon reasonable request.

## Abbreviations

AOR: Adjusted odds ratio
APH: Antepartum Hemorrhage
CI: Confidence Interval
CSA: Central Statistics Agency
EDHS: Ethiopia Demography and Health Survey
FDRE: Federal Democratic Republic of Ethiopia
HFIAS: Household Food Insecurity Access Scale
IPVP: Intimate Partner Violence during Pregnancy
IUFD: Intra-uterine Fetal Death
LBW: Low Birth Weight
MUAC: Mid-Upper Arm Circumference
NMR: Neonatal Mortality Rate
SDG: Sustainable Development Goal
UNICEF: United Nations International Children’s Emergency Fund
WHO: World Health Organization
