# Supplementary material for: Household food insecurity and physically demanding work during pregnancy are risk factors for low birth weight in north Shewa zone public hospitals, Central Ethiopia, 2021: a multicenter cross-sectional study
Source: BMC Pediatr. 2022 Jul 14;22:419. doi: 10.1186/s12887-022-03480-2 (PMC9281010; doi:10.1186/s12887-022-03480-2)
Supplement: Supplementary file 1 — Additional file 1. [file 12887_2022_3480_MOESM1_ESM.docx]

### **Instruments for data collection**

### **English version questionnaires**

| **Part I: socio-demographic characteristics of the respondents** | | | | |
| --- | --- | --- | --- | --- |
|  | Name of the hospital | _________________ | |  |
|  | Date of interview | _____\|_____\| ______ DD \|MM \|YYYY | |  |
|  | Characteristics | Response | | Skip |
|  | Participant code | ______________ | |  |
| 101 | Age of the mother (in completed years) | ______________ | |  |
| 102 | Religion | 1. Orthodox 2. Muslim 3. Protestant 4. Catholic 5. If others specify --------- | |  |
| 103 | Marital status | 1. married/living together 2. divorced/separated 3. widowed 4. single/never married | |  |
| 104 | Place of residence | 1. urban 2. rural | |  |
| 105 | Educational status of the mother | 1. had no formal education 2. Primary education (1-8) 3. Secondary education (9-12) 4. Tertiary (college and above) | |  |
| 106 | Occupation status of the mother | 1. Student 2. Housewife 3. Private employed 4. Government employed 5. Merchant 6. If others specify --------- | |  |
| 107 | Average monthly income of the family(cash) | ---------------------- | |  |
| 108 | Educational status of husband/partner | 1. had no formal education 2. Primary education (1-8) 3. Secondary education (9-12) 4. Tertiary (college and above) | |  |
| 109 | Occupation of husband/partner | 1. Student 2. Private employed 3. Government employed 4. Merchant 5. Farmer 6. If others specify --------- | |  |
| 110 | Your household family size | Number______________ | |  |
| **Part II: obstetrics and reproductive related variables** | | | | |
| 201 | The total number of pregnancies (gravida)? | Gravida: _________ | |  |
| 202 | The total number of births (parity)? | Parity: _____________ | |  |
| 203 | The pregnancy was? | 1. Panned and wanted 2. Unplanned but wanted 3. Unplanned and unwanted | |  |
| 204 | The birth interval between this birth and  The preceding birth? | 1. <24 months   2. <24-48 months  3. >48 months | |  |
| 205 | Have you ever had an abortion? | 1. Yes 2. No | | 207 |
| 206 | The total number of abortions? | Number: __________ | |  |
| 207 | Did you have any history of pre-term  Delivery (≤37wk)? | 1. Yes 2. No | |  |
| 208 | Have you ever used family planning | 1. Yes 2. No | |  |
|  | **Part III:** **Maternal medical factors** | | | |
|  | **Characteristic** | **Answer** | | skip |
| 301 | Have you ever faced pregnancy complications during your recent pregnancy? | 1. Yes 2. No | |  |
| 302 | If yes which one of the following | 1. Antepartum hemorrhage 2. Premature of the rapture of membrane 3. pregnancy-induced Hypertension 4. Nausea and vomiting 5. If other ---------------- | |  |
| 303 | During your recent pregnancy, have you been told that you have developed gestational diabetes mellitus? | 1. Yes 2. No | |  |
| 304 | During your recent pregnancy, did you take Iron tablets? | 1. Yes 2. No | | 311 |
| 305 | During the whole pregnancy, for how many days did you take the Iron tablets? | No of Days: ___________ | |  |
| 306 | During your recent pregnancy, have you been told that you have anemia? | 1. Yes 2. No | |  |
| 307 | Do you have any chronic medical illnesses? | 1. Yes 2. No | |  |
| 308 | If yes Which chronic medical illness, do you have? (more than one response is possible) | 1. Chronic hypertension 2. Diabetes mellitus 3. Heart Disease 4. kidney Disease 5. Others (Specify)_________ | |  |
|  | **Part IV; Maternal nutritional status** | | | |
| 401 | Have you got nutritional counselling during your recent pregnancy? | 1. Yes 2. No | |  |
| 402 | What was your meal frequency within a day before this pregnancy? | __________times | |  |
| 403 | Have you taken additional meals during your current pregnancy? | 1. Yes 2. No | |  |
| 404 | What was your meal frequency within a day during your recent pregnancy? | 1. Once 2. Twice 3. Thrice 4. Four times and above | |  |
| 405 | Did you fast during your last pregnancy? | 1. Yes 2. No | |  |
| 406 | In your recent pregnancy Have you ever used alcoholic beverages (wine, beer, areke, tela, etc.)? | 1. Yes 2. No | |  |
| 407 | Have you ever taken herbal medicine during your current pregnancy? | 1. Yes 2. No | |  |
| 408 | During your recent pregnancy, did  Have you ever Smoke? | 1. Yes 2. No | |  |
| 409 | Did your partner ever Smoke? | 1. Yes 2. No | |  |
|  | **Part VII; Regarding food insecurity condition** |  | |  |
|  | In the past four weeks……………. |  | |  |
| 415 | Did you or any household member worry that your household would not have enough food? | 1. Yes 2. No | |  |
| 416 | Were you or any household member not able to eat the kinds of foods you/he/she preferred because of a lack of resources? | 1. Yes 2. No | |  |
| 417 | Did you or any household member have to eat a limited variety of foods due to a lack of resources? | 1. Yes 2. No | |  |
| 418 | Did you or any household member have to eat some foods that you did not want to eat because of a lack of resources to obtain other types of food? | 1. Yes 2. No | |  |
| 419 | Did you or any household member have to eat a smaller meal than you felt you needed because there was not enough food? | 1. Yes 2. No | |  |
| 420 | Did you or any household member have to eat fewer meals in a day because there was not enough food? | 1. Yes 2. No | |  |
| 421 | Was there ever no food to eat of any kind in your household because of lack of resources to get food? | 1. Yes 2. No | |  |
| 422 | Did you or any household member go to sleep at night hungry because there was not enough food? | 1. Yes 2. No | |  |
| 423 | Did you or any household member go a whole day and night without eating anything because there was not enough food? | 1. Yes 2. No | |  |
|  | **Part V; Regarding physical work during pregnancy** | | | |
| 501 | Which activities have you done during your current pregnancy? | Answer | |  |
|  |  | 1=Yes | 2= No |  |
|  | 1. Daily household chores without help of relative person |  |  |  |
|  | 2. Fetching water with large buckets out of compounds |  |  |  |
|  | 3. Lifting heavy loads (>20kg) |  |  |  |
|  | 4. Chopping woods, cutting grass for cattle feeding |  |  |  |
|  | 5. Washing clothes/utensils for long |  |  |  |
|  | 6. Milking cattle |  |  |  |
|  | 7. Digging potatoes |  |  |  |
|  | 8. Planting seeds |  |  |  |
|  | 9. Removing bran from cereals by pounding |  |  |  |
|  | 10. Standing for longer hours (>3hrs) |  |  |  |
|  | 11. Squatting during routine daily activity |  |  |  |
| 502 | How long does it take to reach the health facility? | 1. Less than 1 hour 2. greater than 1 hour | |  |
| 503 | How long time did you take for rest during pregnancy per day? | 1. <2 hr.  2. >2 hr. | |  |
|  | **Part VI; Regarding intimate partner violence during pregnancy** | | | |
|  | During your current pregnancy had your  Husband/intimate partner ever… |  | |  |
| 601 | Slapped or had something thrown at you that could hurt you? | 1. Yes 2. No | |  |
| 602 | Pushed or shoved you? | 1. Yes 2. No | |  |
| 603 | Hit with a fist or something else that could hurt you? | 1. Yes 2. No | |  |
| 604 | Beaten your abdomen? | 1. Yes 2. No | |  |
| 605 | Choked or burnt you on purpose? | 1. Yes 2. No | |  |
| 606 | Threatened to use or used a gun, knife, or another weapon against you? | 1. Yes 2. No | |  |
|  | **Sexual violence** |  | |  |
|  | During your current pregnancy had your  Husband/intimate partner ever…… |  | |  |
| 607 | Physically forced to have sexual intercourse when you did not want to? | 1. Yes 2. No | |  |
| 608 | Had sexual intercourse when you did not want, because you were afraid of what your partner might do to you? | 1. Yes 2. No | |  |
| 609 | Forced you to do something sexual that you found degrading or humiliating? | 1. Yes 2. No | |  |
|  | **Emotional/psychological violence**  During your current pregnancy had your husband /intimate partner ever…… |  | |  |
| 610 | Insulted or made you feel bad about yourself? | 1. Yes 2. No | |  |
| 611 | Belittled or humiliated in front of other people? | 1. Yes 2. No | |  |
| 612 | Had done things to scare or intimidate her on purpose (e.g., by yelling or Smashing things)? | 1. Yes 2. No | |  |
| 613 | Had threatened to hurt her or someone you cared about? | 1. Yes 2. No | |  |
| 614 | Have you ever attended ANC follow up for your current delivery? | 1. Yes 2. No | | 704 |
| 615 | If yes to Q 614 how many times? | 1. 4 visits and above 2. less than 4 visits | |  |
| 616 | If yes to Q 614 at what months of the current pregnancy, you started ANC? | 1. ________in weeks 2. Don't know/not sure | |  |
|  | **Part VI: Extraction checklist from medical charts** |  | |  |
| 704 | How did she give birth? **That is a mode of delivery** | 1. Vaginal delivery 2. Assisted delivery 3. Cesarean section | |  |
| 705 | Type of pregnancy | 1. Singleton birth 2. multiple births | |  |
| 706 | What was her hemoglobin level during her current pregnancy? | 1. ----------------g/dl | |  |
| 707 | HIV status of the mother (PICT) | 1. Reactive 2. Non-reactive 3. Don’t know | |  |
|  | **Part VII: newborn characteristic from records** | | | |
| 801 | Neonatal birth weight in grams? | In gm: ____________ | |  |
| 802 | Sex of the newborn? | 1. Male 2. Female | |  |
| 803 | Gestational age (GA) at Delivery | _______wks. | |  |
|  | **Measurement** | | | |
| 804 | MUAC (Take left hand if right handed, and right hand if left- handed) | 1. ________cm | |  |
